# Efficient Vaccine Distribution Based on a Hybrid Compartmental Model

**DOI:** 10.1371/journal.pone.0155416

**Published:** 2016-05-27

**Authors:** Zhiwen Yu, Jiming Liu, Xiaowei Wang, Xianjun Zhu, Daxing Wang, Guoqiang Han

**Affiliations:** 1 School of Computer Science and Engineering, South China University of Technology, Guangzhou, Guangdong, China; 2 Department of Computing, Hong Kong Baptist University, Kowloon Tong, Hong Kong; University of Waterloo, CANADA

## Abstract

To effectively and efficiently reduce the morbidity and mortality that may be caused by outbreaks of emerging infectious diseases, it is very important for public health agencies to make informed decisions for controlling the spread of the disease. Such decisions must incorporate various kinds of intervention strategies, such as vaccinations, school closures and border restrictions. Recently, researchers have paid increased attention to searching for effective vaccine distribution strategies for reducing the effects of pandemic outbreaks when resources are limited. Most of the existing research work has been focused on how to design an effective age-structured epidemic model and to select a suitable vaccine distribution strategy to prevent the propagation of an infectious virus. Models that evaluate age structure effects are common, but models that additionally evaluate geographical effects are less common. In this paper, we propose a new SEIR (susceptible—exposed—infectious šC recovered) model, named the hybrid SEIR-V model (HSEIR-V), which considers not only the dynamics of infection prevalence in several age-specific host populations, but also seeks to characterize the dynamics by which a virus spreads in various geographic districts. Several vaccination strategies such as different kinds of vaccine coverage, different vaccine releasing times and different vaccine deployment methods are incorporated into the HSEIR-V compartmental model. We also design four hybrid vaccination distribution strategies (based on population size, contact pattern matrix, infection rate and infectious risk) for controlling the spread of viral infections. Based on data from the 2009–2010 H1N1 influenza epidemic, we evaluate the effectiveness of our proposed HSEIR-V model and study the effects of different types of human behaviour in responding to epidemics.

## Introduction

The rapid spread of an infectious disease can have a devastating impact on human welfare, lowering the quality of people’s lives and causing increased mortality. For example, during the SARS outbreak in 2003, 299 people died, 1,755 persons were infected by the disease in Hong Kong city, and the lives of millions of people were affected [1]–[3]. In the 2009 Hong Kong H1N1 epidemic, 36,896 people suffered from the flu [4]–[5]. The cholera epidemic in Haiti from 2010 to 2011 led to 779,000 cases of the disease and 11,100 deaths [6]–[8].

To prevent an infectious disease from spreading, various epidemic models have been designed to help public health agencies in making strategic decisions. Different intervention strategies, such as vaccination, antiviral prophylaxis and treatment, area or household quarantine or case isolation have been incorporated into various epidemic models. Some researchers have focused on single-focus strategies [9]–[13]. For example, Ferguson et al. [9] studied the effects of various particular strategies on disease control, including border restrictions, internal travel restrictions, school closures, case isolations, household quarantines, treatment of clinical cases, household-based prophylaxis and vaccination. Oles et al. [10] explored strategies such as global preventive treatment, local treatment and palliative treatment to control the propagation of an epidemic and to reduce its total cost. Tang et al. [11] developed community-based intervention strategies such as quarantines, isolations, hygiene precautions, school closures and travel precautions to reduce the effects of the 2009 H1N1 epidemic in China. Jackson et al. [12] explored the effects of school closures on influenza outbreaks.

Other recent research work has emphasised analysing combinations of intervention strategies [14]–[26]. For example, Lee et al. [14] explored the effects of combining different strategies such as vaccinations, antiviral prophylaxis and treatment, area or household quarantines, case isolations, social distancing measures and air travel restrictions for mitigating the effects of an influenza pandemic. These authors concluded that combined strategies could be more effective than individual strategies. Oles et al. [15] considered a balance of strategies focused on treatment and recovery.

Moss et al. [19] integrated both diagnosis and antiviral intervention strategies into the SEIR (susceptible—exposed—infectious šC recovered) model to prevent the propagation of an infectious disease. Zhang et al. [20] adopted a strategy that combined the interventions of workforce shifts and school closures.

Other studies have proposed optimal intervention strategies for situations with limited available resources.

Dimitrov et al. [22] searched for the optimal antiviral strategies for distributing antiviral medications from the U.S. Strategic National Stockpile (SNS) to prevent the transmission of H1N1 in the fall of 2009. These authors also studied the optimal use of the U.S. antiviral SNS to prevent the spread of a pandemic influenza [23]. Wallinga et al. [25] explored an optimal intervention strategy by adjusting the prioritisation of groups for intervention when encountering new observations during an epidemic. In addition, some researchers have investigated how to detect potential epidemics in their early stages [27]–[33].

Recently, many studies have explored different vaccine deployment strategies. These studies have investigated how to identify the optimal approaches for distributing vaccine effectively. Clearly, vaccination is one of the most effective and efficient intervention strategies for reducing morbidity and mortality and for preventing outbreaks of epidemic.

Unfortunately, vaccine stockpiles may not be adequate during the spread of an infectious disease. The possible reasons for shortages of vaccine stockpiles can involve difficulties with the identification of vaccine compositions [34], limitations in manufacturing [35], challenges concerning logistics [35], or economic limitations [36]. To solve these various problems, the World Health Organization (WHO) has recommended that several population groups with high risk of death have priority for vaccination during epidemics in which vaccine resources are limited. The question of how to make the fullest use limited vaccine stockpiles is gaining increasing attention from researchers. For example, as explained in [37], the effectiveness of vaccine distribution is related to three main factors: (1) vaccine coverage (the total number of vaccine doses available), (2) vaccine releasing time (the starting date of vaccine deployment) and (3) vaccine deployment methods (the distribution of vaccine doses to different host populations). Liu et al. [38] designed an age-structured SEIR model to simulate the dynamics of epidemic expansion under various vaccine deployment conditions. These authors evaluated the effects of vaccine deployment factors and identified vaccination priorities for different population groups. Other researchers have sought to identify the priorities for the vaccination of various populations in different regions or cities, rather than for age groups. For example, Araz et al. [39] analysed the geographic spread of influenza and designed a modified SEIR model based on both the populations in different age groups and in different regions. These authors were able to identify a geographic prioritisation-based vaccine distribution strategy.

In general, most existing studies have considered either the spread of specific infectious diseases among different age populations, or among the populations [40] [41] of different geographical regions [42] [43]. Few studies have incorporated both the age factor and the geography factor into an infectious compartmental model. Our previous work in [38] investigated how to model social contacts from census data. In this approach, we sought to capture not only the hierarchical relationships of social contact patterns with respect to the geographic factor, but also to characterise these relationships by considering both age and geography factors. Unfortunately, this study did not take the vaccine distribution strategy into account. Therefore, we feel that developing an adaptive vaccine distribution strategy for host populations in terms of different age groups and different geographic districts is one of the most interesting and important issues for researchers to investigate.

In this paper, we consider to the problem of how to effectively and efficiently distribute vaccines by taking into account the factors of both age and geography. We propose a new SEIR model, named the hybrid susceptible—exposed—infectious—recovered—vaccinated (V) compartmental model (or HSEIR-V for short), to simulate the dynamics of an infectious disease in both age-structured and district-based subpopulations. Different vaccination and segregation strategies are incorporated into the proposed HSEIR-V model to characterise the outbreak of an epidemic. We also carry out some simulation-based experiments to investigate the effects of vaccine coverage, the effects of different releasing times and the effects of different vaccine distribution methods on disease control. We identify an optimal combination of vaccine deployment and segregation strategies. The simulation-based experimental results demonstrate that our proposed HSEIR-V model and the corresponding vaccination and segregation strategies are useful in effectively preventing the spread of disease.

Our paper makes two main contributions. First, we design an extended SEIR epidemic model (named the HSEIR-V model), which takes into account both the age and the geographic district factors and includes both vaccination and the segregation strategies to better simulate the dynamics of epidemics and their treatment. Second, we consider different hybrid vaccination distribution and segregation strategies based on the relevant population size, the contact pattern matrix, the infection rate and the infectious risk. All of these factors are taken into account in our study as we seek to develop more effective strategies for controlling the spread of viral infections.

The remainder of the paper is organised as follows. Section II performs simulation-based experiments to evaluate the performance of our proposed model, and shows the results and discussions. Section III describes the proposed HSEIR-V compartmental model and the corresponding model parameters, and presents different vaccination strategies. Section IV introduces the previous work as related to vaccine distribution strategies. Section V presents our conclusions.

## Results/Discussion

We carry out simulation-based experiments to evaluate the effects of different vaccine distribution strategies in combination with segregation strategies as they relate to the dynamics of disease transmission during the 2009 Hong Kong H1N1 swine-flu epidemic. The parameters adopted in our HSEIR-V model include the population sizes and infection rates *θ*_*ij*_ that correspond to different regions (*R*) and different age groups (*A*), as listed in Tables 1 and 2. The data in Table 1 are extracted from a Hong Kong government report entitled “The Profile of Hong Kong Population Analysed by District Council District” [44]. The data in Table 2 are calibrated based on the infection rates given in [38]. As the infection rate is a biological indicator that differs from the physiological characteristics of individuals (such as their ages, genders and racial identities), this indicator is not sensitive to geographical districts, but is affected by the population structure with respect to people’s ages. As a result, the infection rates shown in Table 2 have the same values with respect to different geographical districts, but they have different values in terms of different age groups. The incubation rates *φ*_*ij*_ and the recovery rates *ψ*_*ij*_ for all of the subpopulations are set to 0.25 and 0.334, respectively. The segregation rate in the exposed compartment, *α*_*ij*_, the segregation rate in the infectious compartment, *β*_*ij*_, and the treatment rate, *γ*_*ij*_, for the segregation of individuals for all of the subpopulations are set to 0.06, 0.03 and 0.7, respectively. The basic reproduction rate, *R*_0_, is set to 1.2 according to the research work by Cowling [45].

**Table 1:** The subpopulation sizes (10^6^) with respect to different districts (*R*) and different age groups (*A*).

	*A*_1_ (5–14)	*A*_2_ (15–24)	*A*_3_ (25–44)	*A*_4_ (45–64)	*A*_5_ (65+)
*R*_1_(Hong Kong Island)	0.1468	0.1468	0.4187	0.3932	0.1711
*R*_2_(Kowloon Peninsula)	0.2404	0.2465	0.6323	0.5939	0.3071
*R*_3_(New Territories)	0.4758	0.5154	1.1714	1.0885	0.3532

**Table 2:** The infection rates *θ*_*ij*_ with respect to different districts (*R*) and different age groups (*A*).

	*A*_1_ (5–14)	*A*_2_ (15–24)	*A*_3_ (25–44)	*A*_4_ (45–64)	*A*_5_ (65+)
*R*_1_(Hong Kong Island)	0.434	0.158	0.118	0.046	0.046
*R*_2_(Kowloon Peninsula)	0.434	0.158	0.118	0.046	0.046
*R*_3_(New Territories)	0.434	0.158	0.118	0.046	0.046

Table 3 shows the parameter settings for vaccine distribution (where |*P*| denotes the size of the population), which includes the default values and ranges. We vary the values of the parameters one at a time in the experiments that follow, while setting the values of the other parameters to their default values. During the 2009 Hong Kong H1N1 swine-flu epidemic, the Centre for Health Protection (CHP) of Hong Kong prepared 0.5 × 10^7^ vaccine doses for the citizens, which was nearly 10% of the total population |*P*| in our study. The default value of vaccine coverage was set to *O*_2_ = 0.04|*P*|. As the beginning of the spread of the infectious virus was from 50 days during the H1N1 influenza epidemic in Hong Kong in 2009, the default setting for the vaccine releasing time is set to *T*_2_ = 50 days. The default settings of vaccine distribution strategies are set to S1. One possible reason for this strategy is that S1 is the most popular strategy, as this approach reduces any grounds for controversy among local authorities.

**Table 3:** The parameter settings for vaccine distribution (where |*P*| denotes the size of the population).

Parameters	Default value	Range
Vaccine coverage	*O*_2_ = 0.04|*P*|	*O*_1_ = 0.02|*P*|, *O*_2_ = 0.04|*P*|, *O*_3_ = 0.06|*P*|, *O*_4_ = 0.08|*P*|, *O*_5_ = 0.1|*P*|
Vaccine releasing time	*T*_2_ = Day 50	*T*_1_ = Day 1, *T*_2_ = Day 50, *T*_3_ = Day 100, *T*_4_ = Day 150, *T*_5_ = Day 200
Vaccine distribution strategies	*S*1	*S*1, *S*2, *S*3, *S*4

We adopt two model outcome measures to evaluate the effects of vaccine distribution strategies. These measures include (1) the final infection attack rates, according to the number of accumulated individuals and (2) the size of the infectious population during the spread of the virus. Our simulation is based on several assumptions concerning the effectiveness of vaccination. First, all of the vaccinated individuals gain complete immunisation. Second, the susceptible individuals are protected once they are vaccinated. Third, all of the individuals are willing to be vaccinated.

In the following experiments, we first simulate the dynamics of the infectious virus and the spread of infectious virus without vaccination. Then, we consider the effects of vaccine coverage and vaccine releasing time. Following this, we explore different vaccine distribution strategies for controlling the dynamics of the epidemic. Last, we study the effects of the vaccination strategy in combination with the segregation strategy.

### Simulation of dynamics of infectious virus

**Fig 1:**
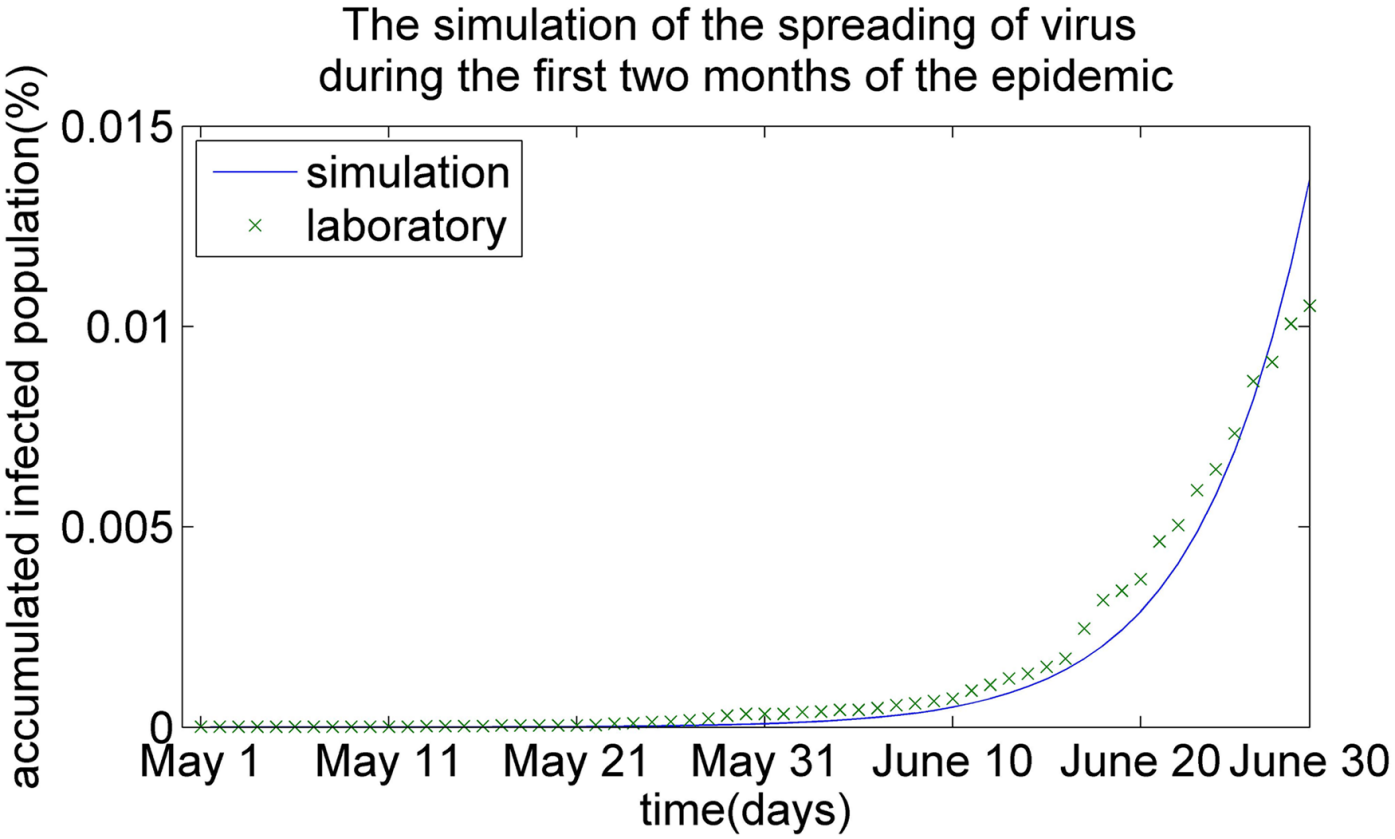
The simulation of the virus’s spread during the first two months of the epidemic.

We first evaluate the effectiveness of the HSEIR-V model to simulate the spread of infectious virus during the 2009 Hong Kong H1N1 swine-flu epidemic. When the first infectious case was confirmed by the Centre for Health Protection (CHP) of Hong Kong on May 2, 2009, the public health department proposed several intervention strategies, such as the vaccination strategy, the segregation of infection cases and school closures to control the dynamics of the epidemic. The circular line in Fig 1 shows the number of infectious cases confirmed in the laboratory by CHP in practice during the first two months of the epidemic following May 2, 2009.

The HSEIR-V model adopts the above-mentioned model parameters to simulate the dynamics of the H1N1 epidemic in Hong Kong with respect to the size of the infectious population. The solid line in Fig 1 illustrates the simulation results obtained by the HSEIR-V model during the period from May 2, 2009 to March 30, 2010 for the Hong Kong H1N1 swine-flu epidemic. It is observed that the two curves in Fig 1 are close to each other, which means that the simulation results obtained by the HSEIR-V model are consistent with the observed dynamics of the infectious virus in practice. One possible reason for this convergence is that the HSEIR-V model takes into account both the geographic district factor and the age factor, which makes this model more truly reflect the spread of the infectious virus. The differences between the simulation results and the laboratory-confirmed cases are caused by the estimated frequency of contacts between different age groups and different districts and the intervention strategies adopted by the CHP of Hong Kong. In general, the HSEIR-model is qualified for simulation of the epidemic’s dynamics, and it is found suitable to explore the effects of different vaccine distribution strategies.

### The spreading of infectious virus without vaccination

**Fig 2:**
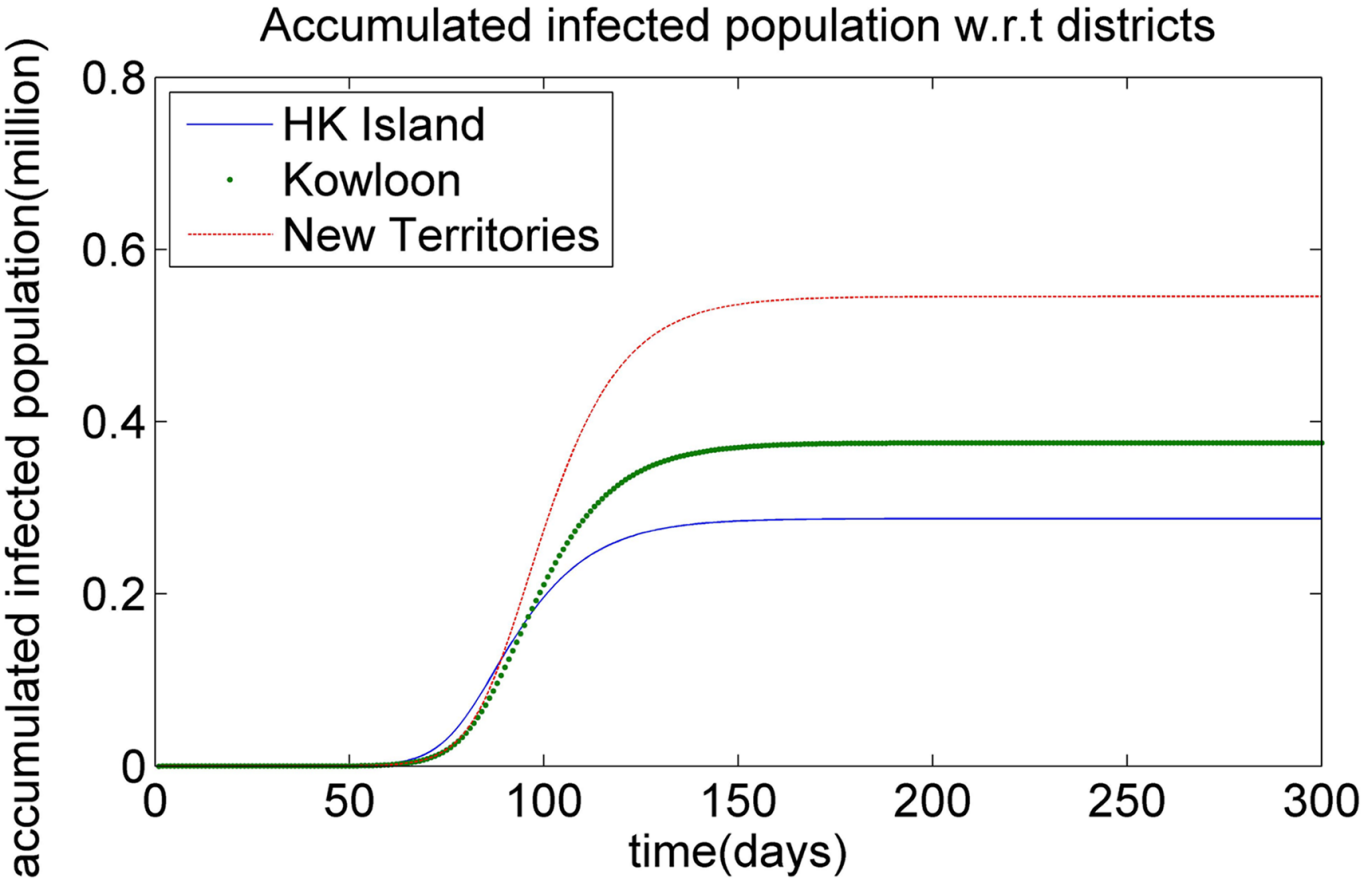
The dynamics of infectious virus with respect to the accumulated infected population for different districts.

**Fig 3:**
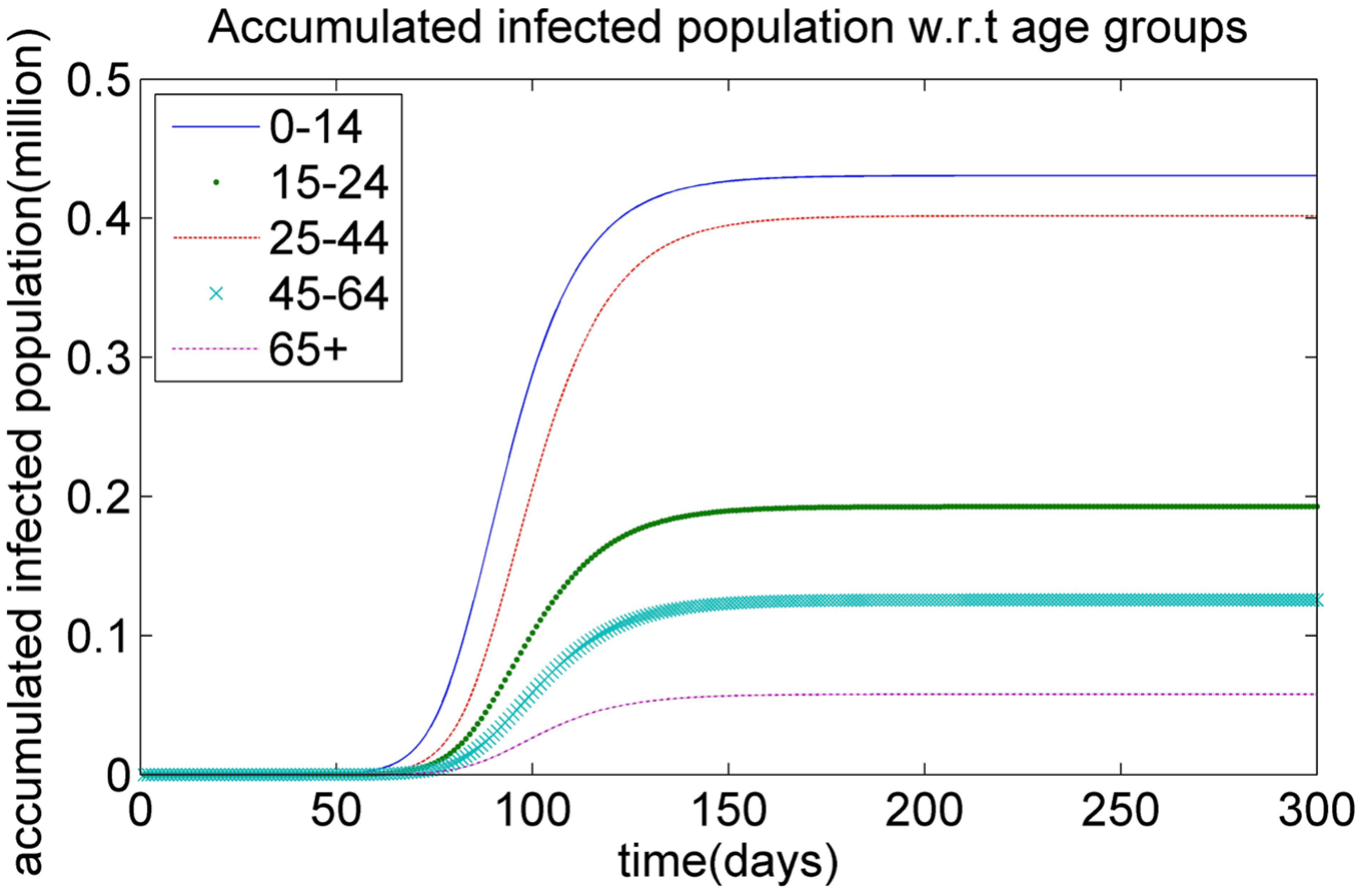
The dynamics of infectious virus with respect to the accumulated infected population for different age groups.

To investigate the spread of the infectious virus without vaccination, we assume that the epidemic begins with a single infectious individual with respect to *A*_3_ age group in New Territories region *R*_3_ in Hong Kong. Figs 2 and 3 show the simulated curve for the dynamic of the H1N1 epidemic in Hong Kong with respect to the accumulated infected population in different regions and different age groups, respectively, during the spread of the infectious virus in the scenario with an initial case in the New Territories region. It can be seen that (1) the accumulated infected population of the age group *A*_1_ is higher than that of other age groups at the earlier stages of the epidemic, as shown in Fig 3. The possible reasons for this development are as follows: (*i*), the first infectious individual at the initial point belongs to the age group *A*_3_. The contact frequency of the individuals between the age groups *A*_1_ and *A*_3_ is high, and the contact frequency of the individuals in the same age group *A*_1_ is also high. The high contact frequency among the individuals in age group *A*_1_ makes it easier for them to be infected when the epidemic breaks out. (*ii*) The infection rate for the age group *A*_1_ is much larger than that for the other age groups. This pattern means that the children in *A*_1_ have low immunity, which leads to a high infectious rate. Once the adults in age group *A*_3_ are infected, the children also have a high probability of being infected, which leads to the larger accumulated infected population of the age group *A*_1_ in the early stage of the epidemic.

(2) The accumulated infected population of the age group *A*_3_ rises quickly during the period of the epidemic, as illustrated in Fig 3. A possible reason for this development is that the individuals in age group *A*_3_ come into contact more easily than the individuals in other age groups. The accumulated contact frequency between age group *A*_3_ and the other age groups (including *A*_3_ itself) for the same region is 17.908, which is higher than that of the other age groups. The higher accumulated contact frequency of age group *A*_3_ leads to the increase of the infected population during the process of the epidemic.

(3) The accumulated infected population of age group *A*_5_ remains at a low level during the period of the epidemic, as shown in Fig 3. A possible reason for this low level is that most of the older peoples prefer to stay home, and hence reduce their frequency of contact with other individuals, which leads to the low infection risk during the outbreak of the infectious disease.

### The effect of vaccine coverage and vaccine releasing time

**Fig 4:**
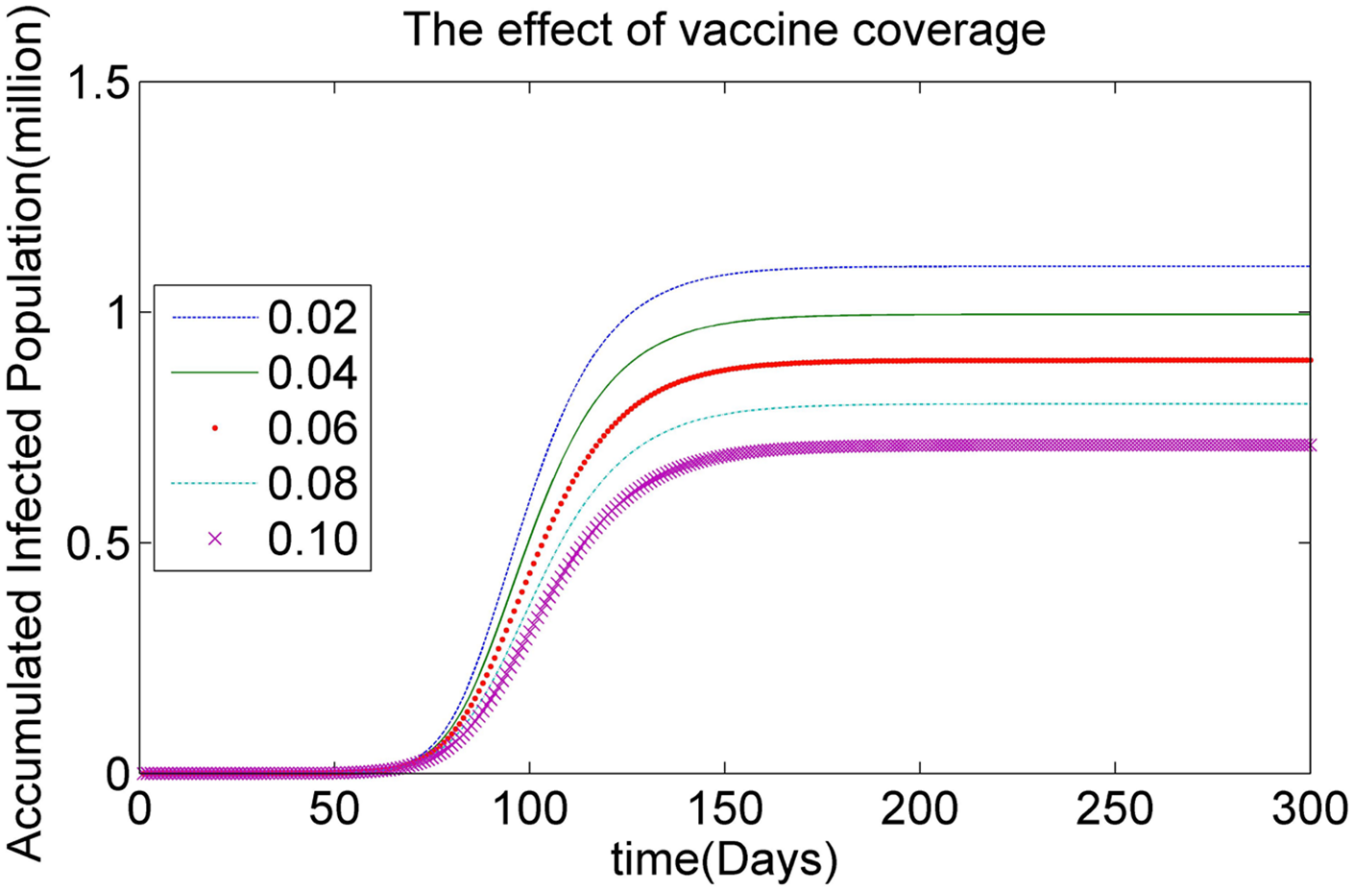
The effect of vaccine coverage.

To study the effect of vaccine coverage *O*, which is the number of vaccine doses, we vary *O* from 0.02 of the total population |*P*| to 0.1 of total population |*P*|, with an increment 0.02|*P*|, as shown in Table 3. Fig 4 shows the simulation results based on the HSEIR-V model with respect to different levels of vaccine coverage. It can be observed that when the number of vaccine doses increases gradually from 0.02|*P*| to 0.1|*P*|, the size of the accumulated infected population decreases gradually, as is illustrated in Fig 4, because more vaccine doses provide antiviral immunisation for more of the vaccinated population.

**Fig 5:**
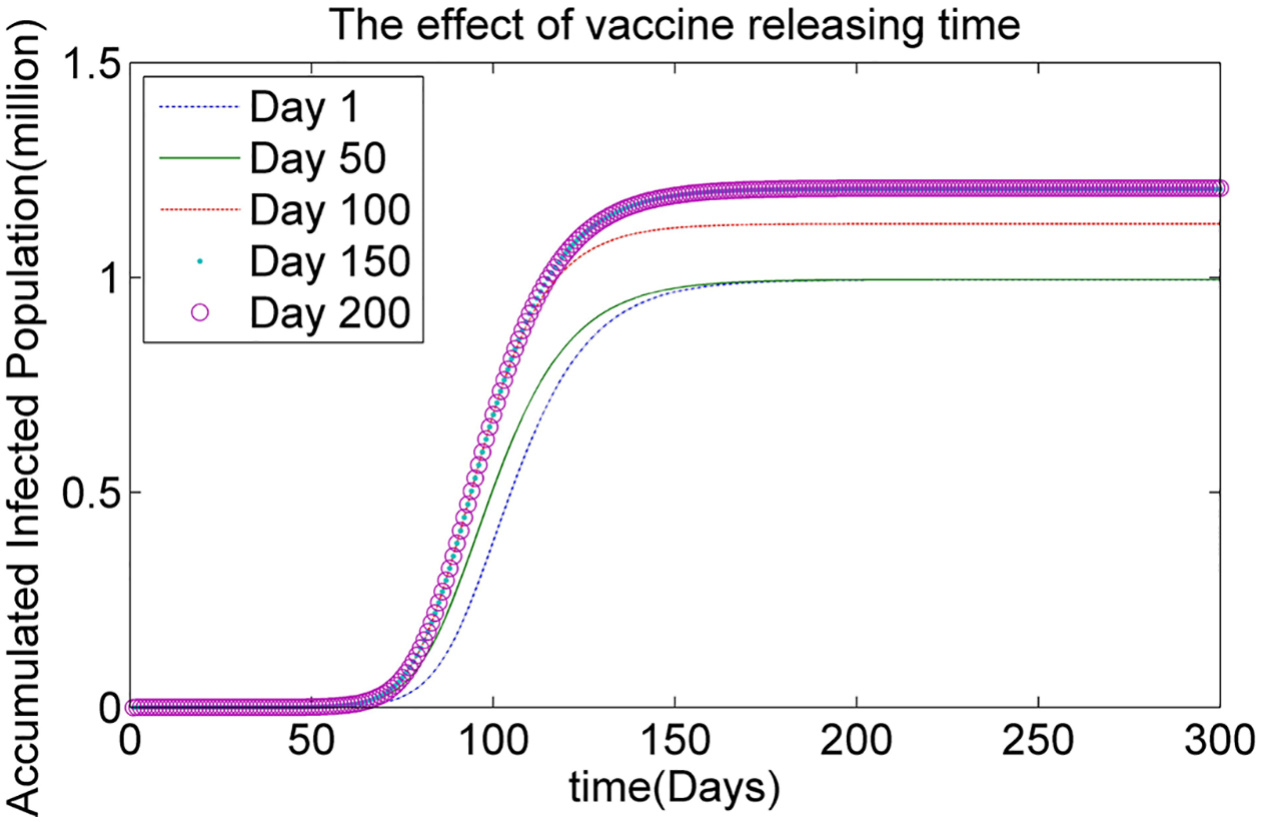
The effect of vaccine releasing time.

To investigate the effects of the vaccine releasing times, we set the vaccine releasing time *T* to Day 1, Day 50, Day 100, Day 150 and Day 200, respectively, as shown in Table 3. Fig 5 illustrates the simulated results based on the HSEIR-V model for the dynamics of the epidemic with respect to the accumulated infected population using different vaccine releasing times. There are several interesting observations to be made concerning these figures. (1) If vaccine doses are released at earlier times in the epidemic, such as Day 1 or Day 50, the size of the accumulated infected population reduces quickly. (2) However, the accumulated infected populations corresponding to the releasing times of Day 150 and Day 200 are similar. This result indicates that if the vaccine doses are released at a later time in the epidemic, the effects of vaccination are limited. In general, the earlier the vaccine releasing time, the smaller the size of the accumulated infected population.

**Fig 6:**
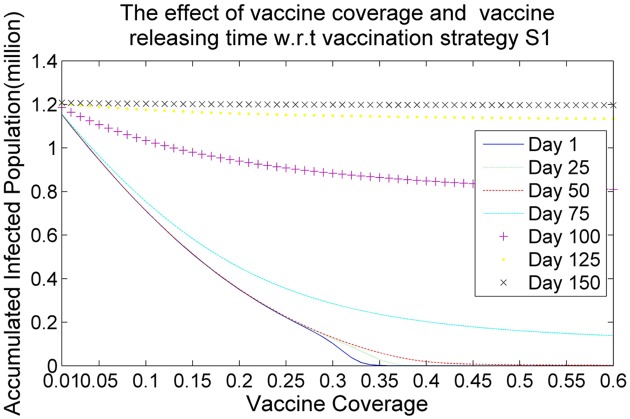
The effects of vaccine coverage and vaccine releasing times with respect to the vaccination strategy *S*1.

**Fig 7:**
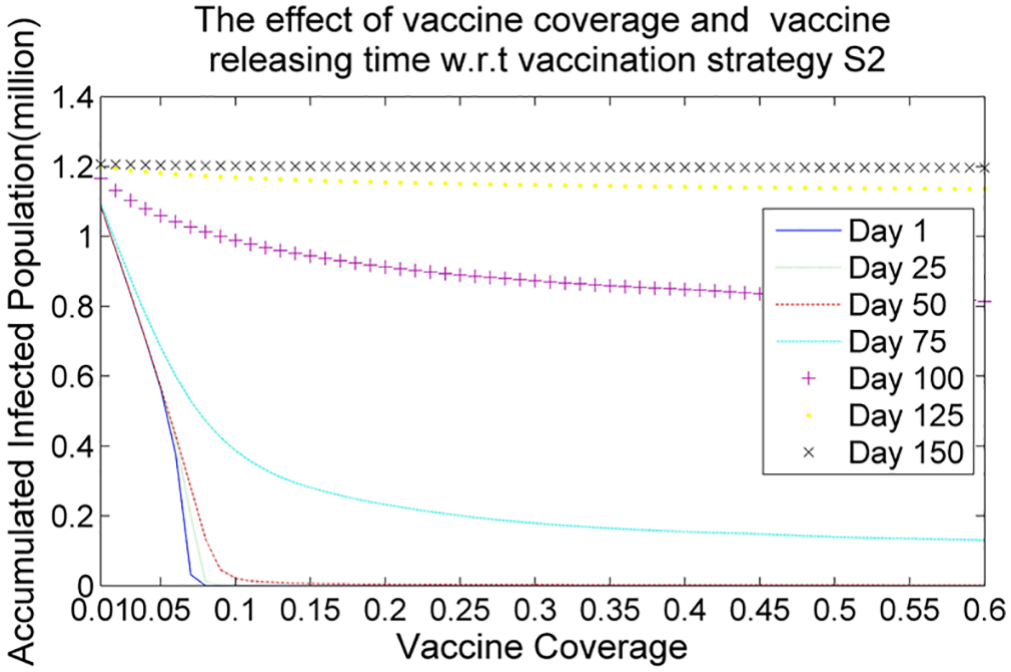
The effect of vaccine coverage and vaccine releasing time with respect to the vaccination strategy *S*2.

**Fig 8:**
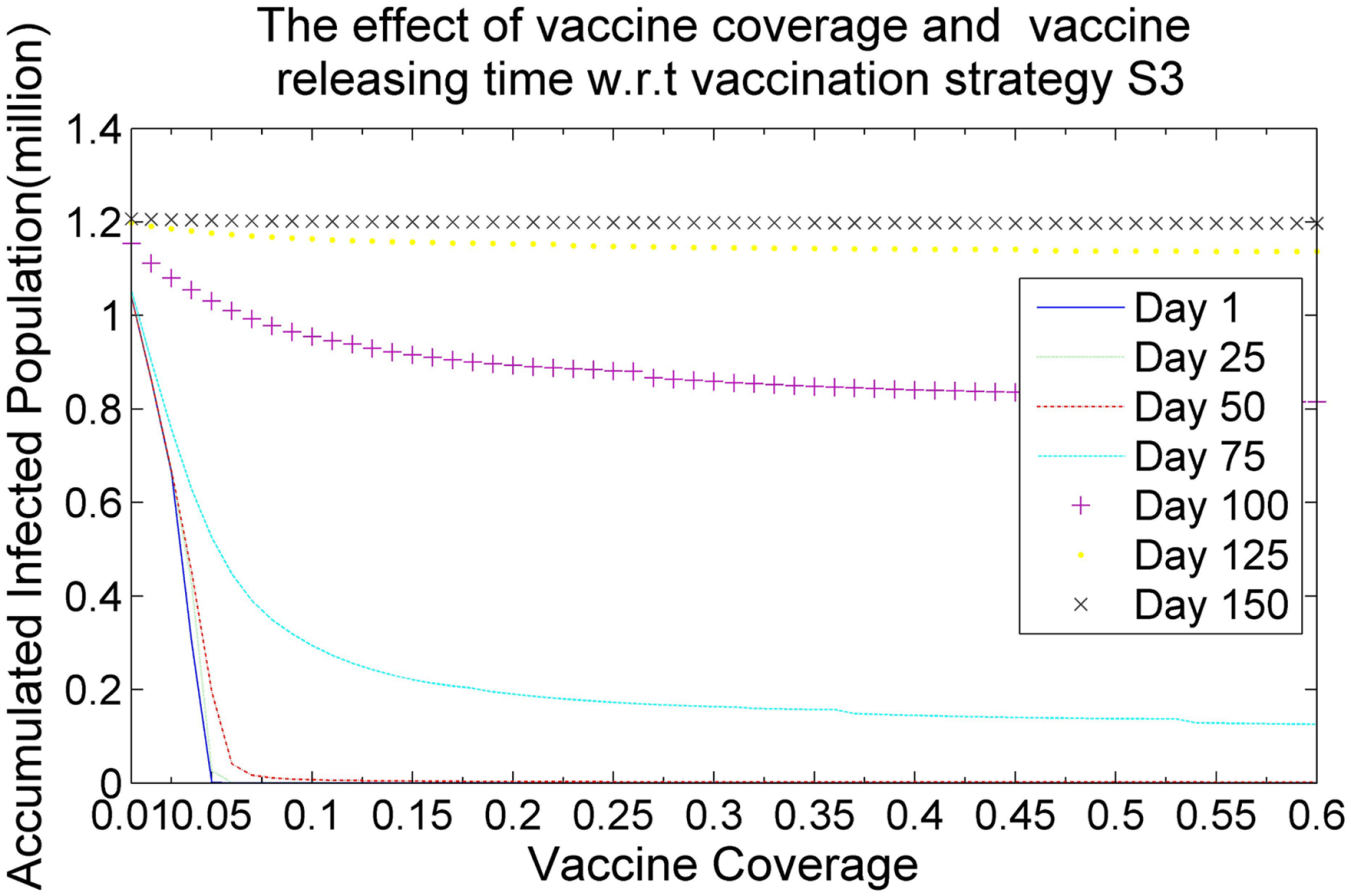
The effects of vaccine coverage and vaccine releasing time with respect to the vaccination strategy *S*3.

**Fig 9:**
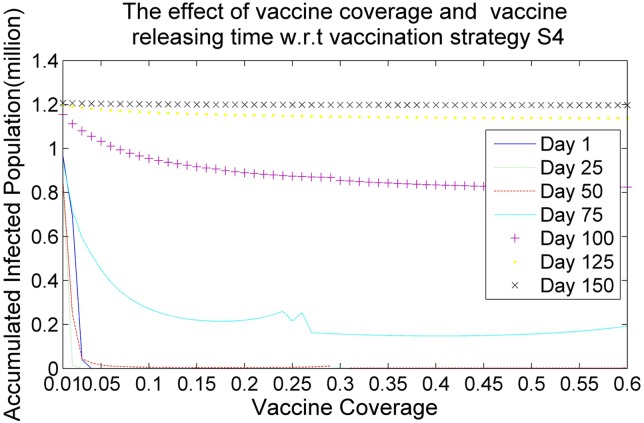
The effects of vaccine coverage and vaccine releasing time with respect to the vaccination strategy *S*4.

Figs 6, 7, 8 and 9 demonstrate the effects of vaccine coverage and vaccine releasing time with respect to the vaccination strategies S1, S2, S3 and S4, respectively. The values of the vaccine coverage vary from 0.01|*P*| to 0.6|*P*| with an increment 0.05|*P*|, but the values of the vaccine releasing times vary from Day 1 to Day 150 with increments of 25 days. There are several interesting observations to be made concerning these results. (1) More vaccine coverage and earlier vaccine releasing time tends to prevent the outbreak of the epidemic. When the number of vaccine doses is larger than 0.1|*P*| and the vaccine releasing time is prior to Day 75, the size of the accumulated infected population drops rapidly, as illustrated in Figs 6–9. (2) The more suitable vaccination strategies, such as *S*4, require a smaller number of vaccine doses to reach herd immunity. If vaccination strategy S4 is adopted and the vaccine doses are distributed at an earlier stage in the spread of the infectious virus (such as Day 1, Day 25 or Day 50) only 0.05|*P*| vaccine doses are required to prevent the outbreak of the epidemic.

### The effect of vaccine distribution strategies

We also study the effect of various vaccine distribution strategies, such as strategy *S*1 (based on population size), strategy *S*2 (based on the contact pattern matrix), strategy *S*3 (based on the infection rate) and strategy *S*4 (based on the infection risk), on the spread of infectious virus with respect to the accumulated infected population.

**Fig 10:**
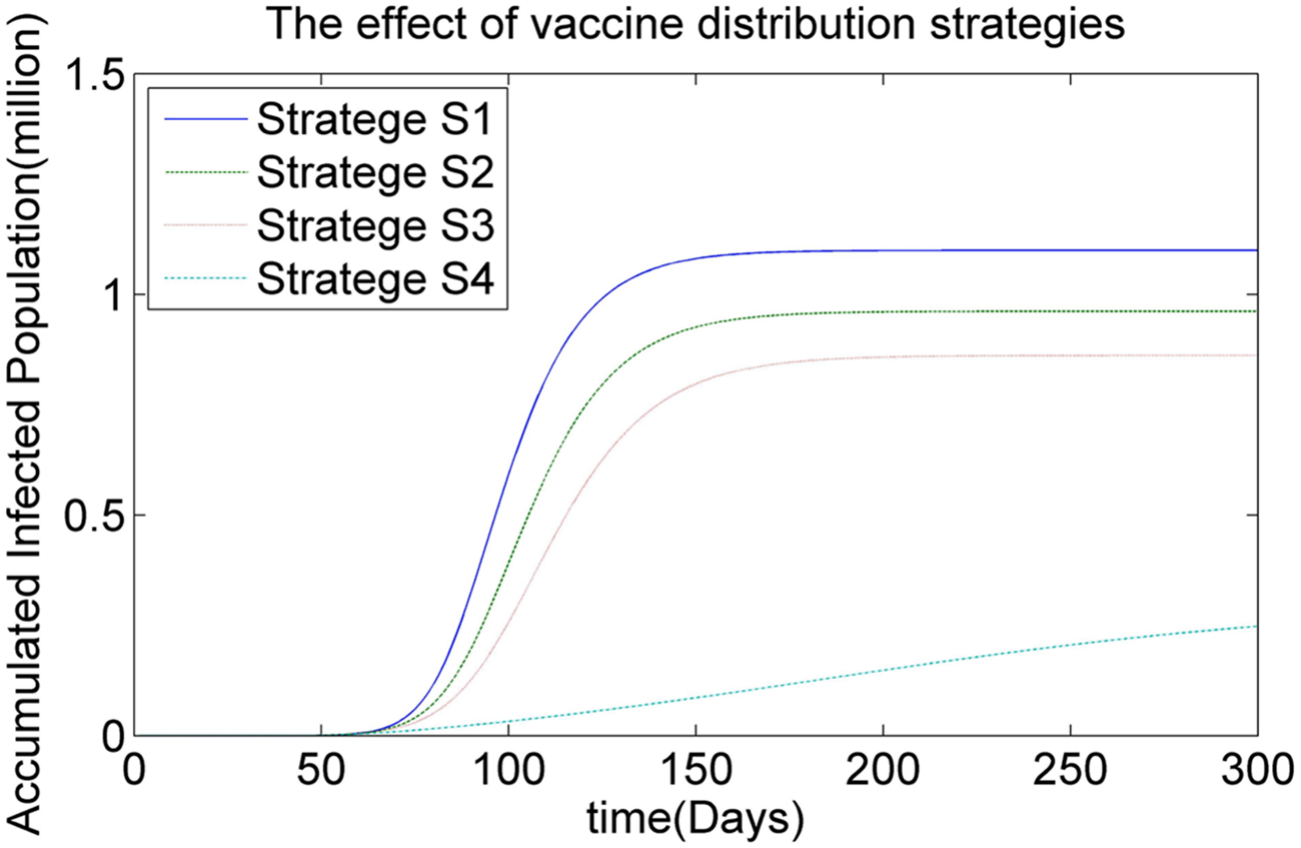
The effects of different vaccination strategies.

**Fig 11:**
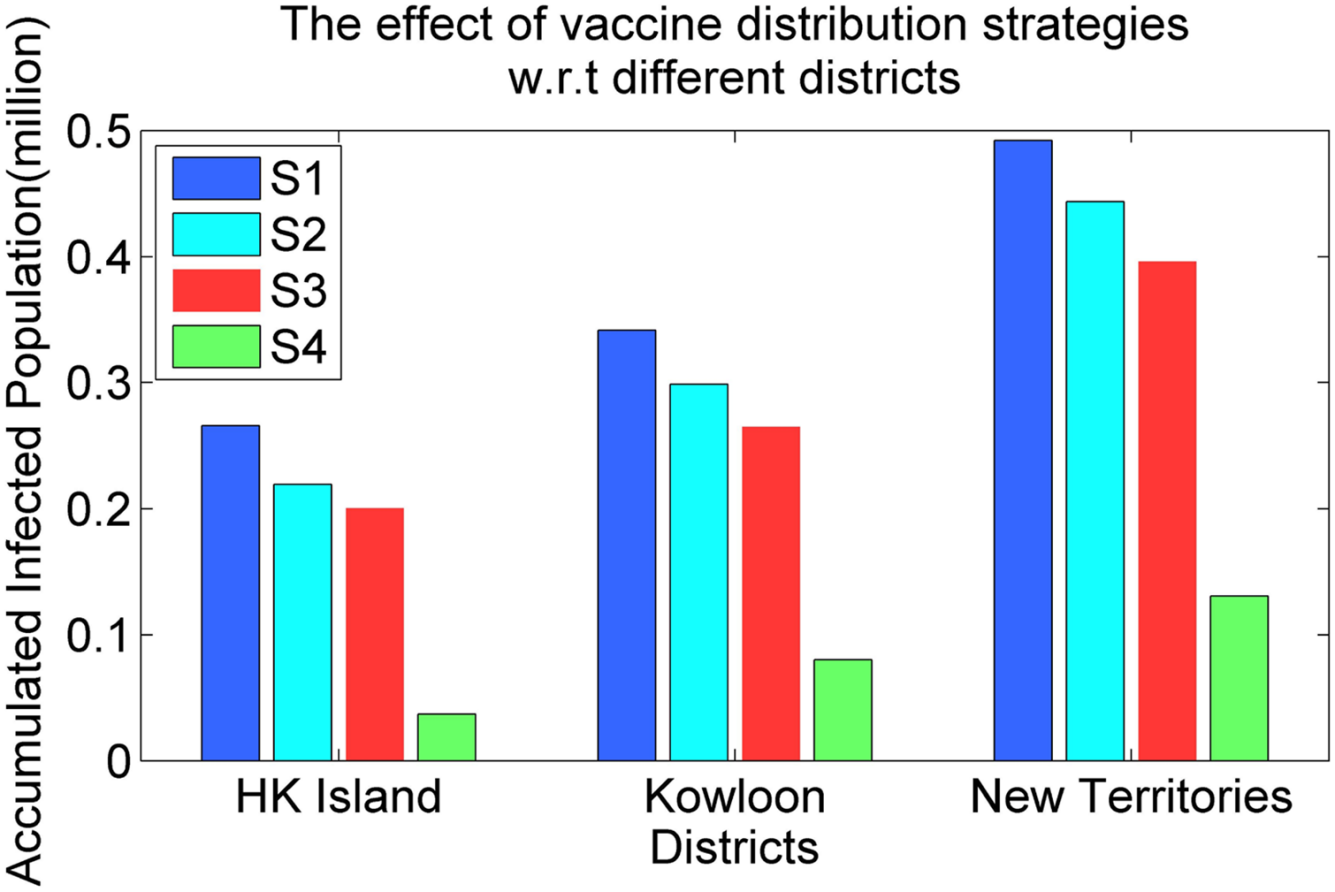
The effects of different vaccination strategies.

Fig 10 illustrates simulated results for the dynamics of the infectious virus with respect to the accumulated infected population for different vaccination strategies in Hong Kong. Fig 11 shows the effect of different vaccination strategies with respect to different districts, such as Hong Kong Island, Kowloon Peninsula and New Territories. As is shown in Figs 10 and 11 (1) the size of the accumulated infected population obtained by vaccination strategy *S*4 is much smaller than that obtained by the other strategies. A possible reason for this result is as follows: vaccination strategy *S*4 focuses on the infection risk *ϕ*_*ij*_, which takes into account both the infection rate and the contact frequencies between individuals. (2) The performance of vaccination strategy *S*1 is the worst among all of the vaccination strategies, as illustrated in Figs 10 and 11. Although *S*1 is able to minimise contradictions among local authorities, it fails to satisfy the real requirement for vaccine doses, which leads to the poor performance of this strategy. (3) The performances of vaccination strategies *S*2 and *S*3 are better than that of *S*3 and worse than that of *S*4. Strategies *S*2 and *S*3 both maintain a balance between the real requirements for vaccine doses and the fair properties with respect to the sizes of population in different regions. As a result, vaccination strategy *S*4 is the best choice when considering the real requirements for vaccine doses.

### The effect of the combination of intervention strategies

**Fig 12:**
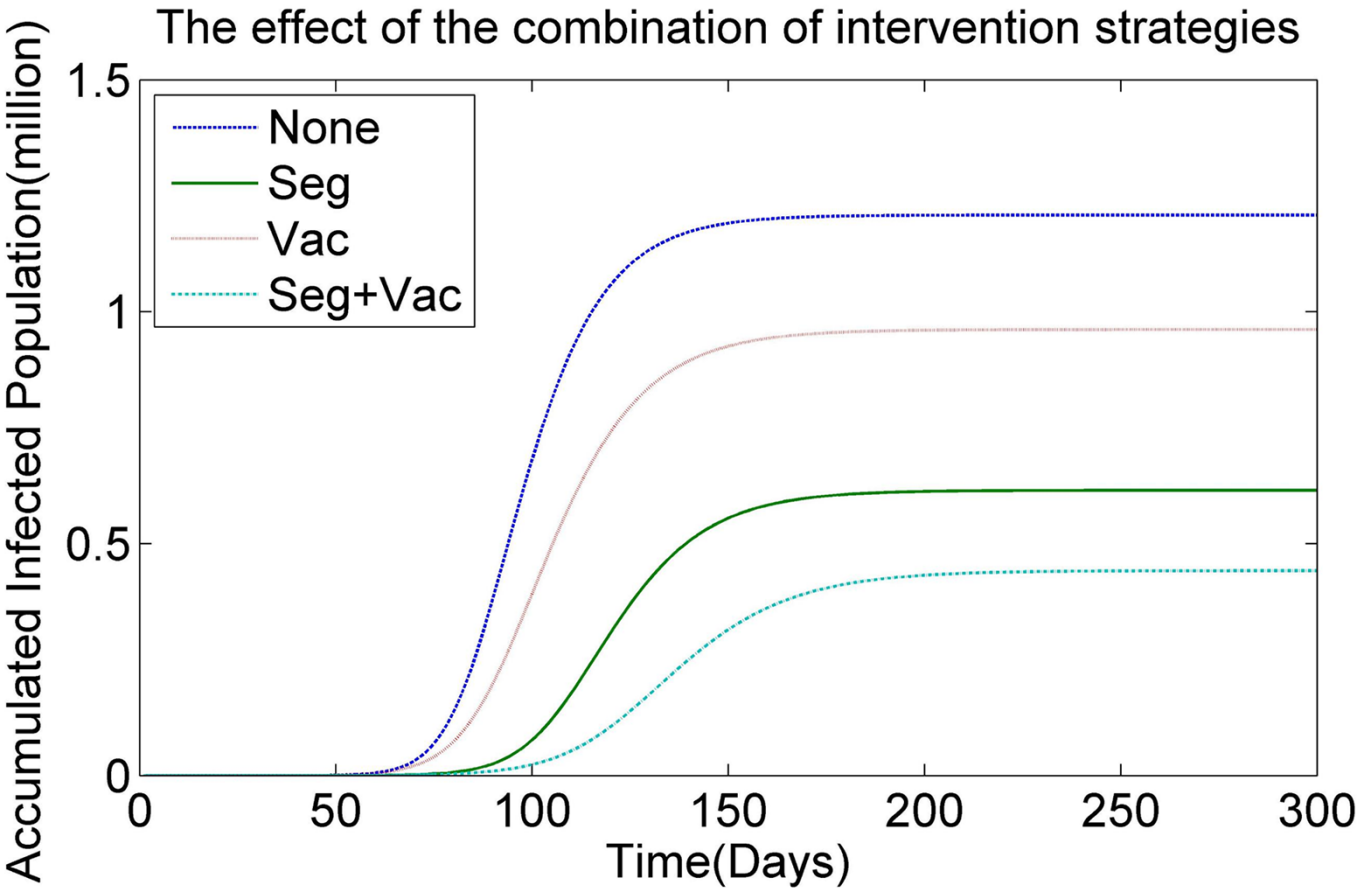
The effect of the combination of intervention strategies.

To explore the effects of the combined intervention strategies, four kinds of intervention strategies are integrated into the HSEIR-V model in our simulations. These four strategies involve doing without an intervention strategy (no strategy), the segregation strategy *Seg*, the vaccination strategy *Vac* and a combination of the vaccination and the segregation strategies, *Seg* + *Vac*. Fig 12 illustrates the simulated results obtained by the HSEIR-V model without any intervention strategy and the HSEIR-V models with strategies *Seg*, *Vac* and *Seg* + *Vac*, respectively. It can be observed that (1) the accumulated infected population obtained by the HSEIR-V model with both strategy *Seg* and strategy *Vac* is better than that obtained by the HSEIR-V model without any intervention strategy. This result indicates that both the vaccination strategy and the segregation strategy are useful for preventing the spread of infectious virus. (2) The segregation strategy is more effective than the vaccination strategy, as the accumulated infected population obtained by the HSEIR-V model with strategy *Seg* is smaller than that obtained by the HSEIR-V model with strategy *Vac*. (3) When compared with the single intervention strategies such as the vaccination strategy or the segregation strategy, the combination strategy *Seg* + *Vac* is more effective, as this approach is able to make a large reduction in number of infected people, as shown in Fig 12. In summary, to reduce the outbreak of an epidemic, the public health department of the government should perform several intervention strategies simultaneously.

## Methods

To capture the dynamics by which viruses spread in different populations, Kermack and McKendrick first proposed the Susceptible šC Infectious—Recovered (SIR) model in 1927, which simulated the dynamics of epidemics [46]. After the first SIR model was developed, various kinds of epidemic models [47]–[49], such as the SEIR model, the MSIR model and the SIS model were designed to help public health agencies in their decision-making tasks. Compared with the SIR model, the SEIR model incorporates exposed compartment E to simulate those individuals who have been infected but are not yet infectious themselves. The MSIR model considers compartment M for maternally derived immunity, and the SIS model takes into account only a distinction between susceptible individuals and infectious individuals. As age is one of the most important factors that affect epidemic models, age-structured epidemic models have gained increasing attention in recent years. However, the dynamic spread of an infectious disease is affected not only by the age factor, but also by the geographic factor.

**Fig 13:**
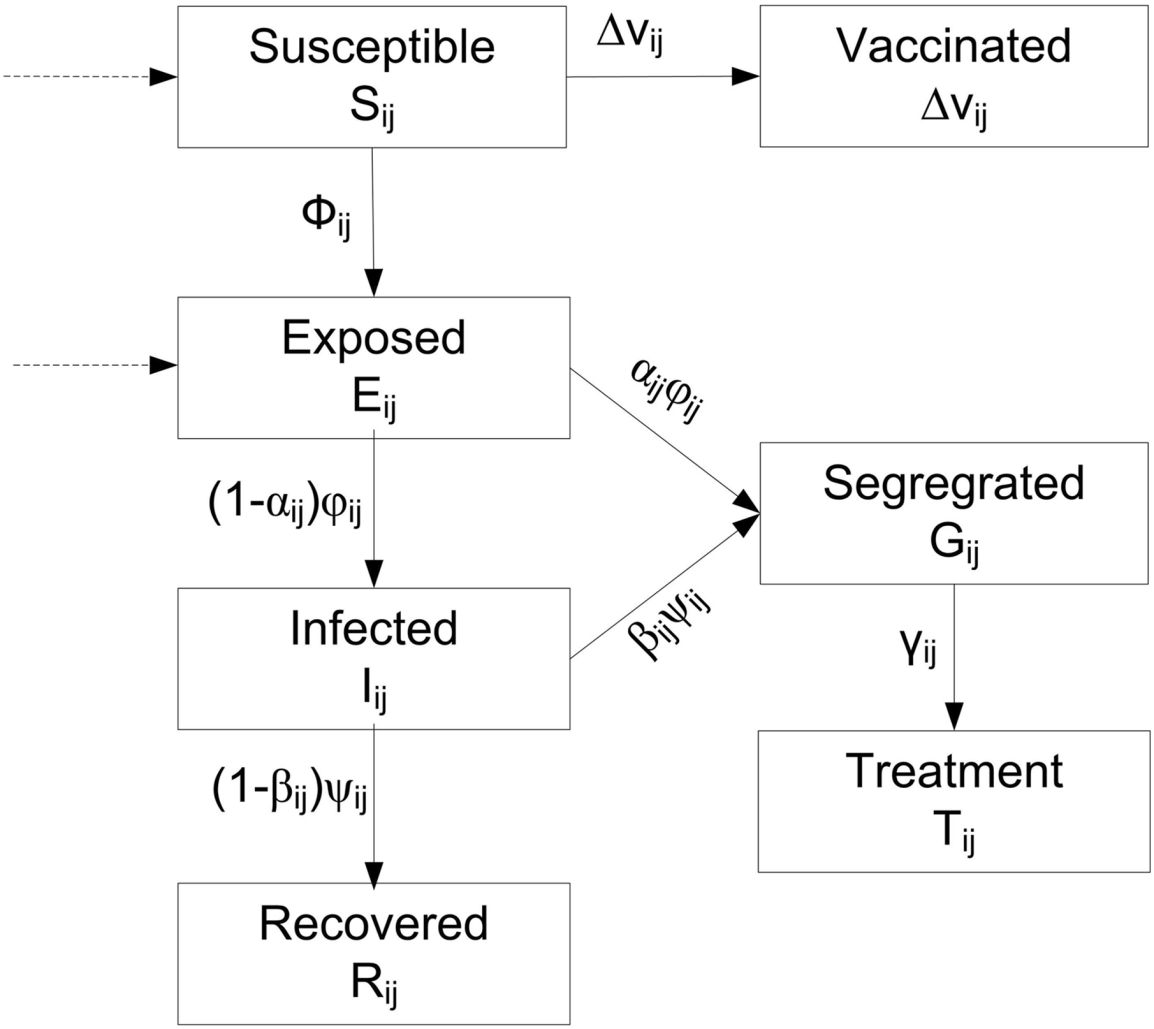
The overview of the proposed epidemic compartmental model.

In this paper, we develop a vaccination-based hybrid SEIR epidemic compartmental model (HSEIR-V) which takes into account both the age and the geographic district factors. We apply this model to simulate the 2009 Hong Kong H1N1 epidemic. Compared with traditional SEIR models, the HSEIR-V model takes into account multiple districts with different host population groups. For example, Hong Kong City is divided into three main districts, namely Hong Kong Island, Kowloon Peninsula and the New Territories. The population in each district is further divided into five age groups, which are A1 (ages 5–14), A2 (15–24), A3 (25–44), A4 (45–64) and A5(65+). The dynamic spread of a disease in each district *i* for each age group *j* is modelled by a modified SEIR model, which consists of several compartments as illustrated in Fig 13: Susceptible *S*_*ij*_, Exposed *E*_*ij*_, Infected *I*_*ij*_, Recovered *R*_*ij*_, Vaccinated *V*_*ij*_, Segregrated *G*_*ij*_ and Treated *T*_*ij*_. The dynamics of virus compartmental infection are defined as follows:

$$\frac{dS_{ij}}{dt}=-\phi_{ij}\cdot \left[\Gamma \left(S_{ij}\right)-\Delta v_{ij}\right]+\left(-\Delta v_{ij}\right)\;\;\;\;\;\;\;$$ (1)

$$\frac{dE_{ij}}{dt}=-\varphi_{ij}\cdot \Gamma \left(E_{ij}\right)+\phi_{ij}\cdot \left[\Gamma \left(S_{ij}\right)-\Delta v_{ij}\right]\;\;\;$$ (2)

$$\frac{dI_{ij}}{dt}=-\psi \cdot I_{ij}+\varphi_{ij}\cdot \left(1-\alpha_{ij}\right)\cdot \Gamma \left(E_{ij}\right)\;\;\;\;\;\;\;$$ (3)

$$\frac{dR_{ij}}{dt}=\psi \cdot \left(1-\beta_{ij}\right)\cdot I_{ij}$$ (4)

$$\frac{dV_{ij}}{dt}=V_{ij}$$ (5)

$$\frac{dG_{ij}}{dt}=\varphi_{ij}\cdot \alpha_{ij}\cdot \Gamma \left(E_{ij}\right)+\psi \cdot \beta_{ij}\cdot I_{ij}-\gamma_{ij}\cdot G_{ij}$$ (6)

$$\frac{dT_{ij}}{dt}=\gamma_{ij}\cdot G_{ij}$$ (7)

where the summary of the definition of parameters is shown in Table 4. Δ*v*_*ij*_ denotes the vaccination rate.

**Table 4:** The summary of the definition of parameters.

Parameters	Definition
*ϕ*_*ij*_	Infection risk
*φ*_*ij*_	Incubation rate
*ψ*_*ij*_	Recovery rate
*α*_*ij*_	Segregation rate in the exposed compartment
*β*_*ij*_	Segregation rate in the infectious compartment
*γ*_*ij*_	Treatment rate for the segregation individuals
Δ *v*_*ij*_	Vaccination rate

The Γ is used to simulate the interaction between different districts, which is based on the commute pattern between districts. Assume that R denotes the set of districts, and that M denotes a symmetric matrix [39] whose entries, *m*_*ih*_ (*i*, *h* ∈ |*R*|, where |*R*| denotes the number of districts), are defined by daily commuting patterns between regions, which are measured as the total number of individuals commuting from the *i*th district to the *l*th district. Γ is a commuting operator defined on the Susceptible and Exposed compartments as shown in Fig 13 (where the dotted line arrow denotes the interaction between different districts). Specifically, the populations in the Susceptible and Exposed compartments include the individuals who come from other districts, and the individuals who go to other districts are removed.

$$\Gamma \left(S_{ij}\right)=S_{ij}+\Sigma_{h\in |R|}\left(m_{hi}\cdot \frac{S_{hj}}{N_{hj}}\right)-\Sigma_{i\in |R|}\left(m_{ih}\cdot \frac{S_{ij}}{N_{ij}}\right)$$ (8)

$$\Gamma \left(E_{ij}\right)=E_{ij}+\Sigma_{h\in |R|}\left(m_{hi}\cdot \frac{E_{hj}}{N_{hj}}\right)-\Sigma_{i\in |R|}\left(m_{ih}\cdot \frac{E_{ij}}{N_{ij}}\right)$$ (9)

It is reasonable to assume that the size of the input Susceptible and Exposed populations and the size of the output Susceptible and Exposed populations maintain a balance within each geographical district of the city in our simulation.

The HSEIR-V epidemic compartmental model takes into account both the risk of infectious contacts with respect to heterogeneous population groups of different ages and different districts and the infection rate *θ*_*ij*_ (generic infection vulnerability), which is defined as follows:

$$\phi_{ij}=\frac{1}{\left|R||A\right|}\left(\sum_{h=1}^{\left|R\right|}\sum_{k=1}^{\left|A\right|}c_{\left((i-1)*|A|+j,(h-1)*|A|+k\right)}\;\cdot \frac{I_{hk}}{P_{hk}}\right)\cdot \frac{S_{ij}}{P_{ij}}\cdot \theta_{ij}$$ (10)

where *ϕ*_*ij*_ is adopted to represent the dynamics of an infectious disease among heterogeneous populations with respect to different districts (*R*) and different age groups (*A*). This variable denotes the probability that susceptible individuals in the population *P*_*ij*_ in the *i*-th district and the *j*-th age group will be infected at the current time during the process of the epidemic. |*R*| is the number of districts, |*A*| is the number of age groups, and *I*_*hk*_ and *P*_*hk*_ denote the infectious individuals in the population in the *h*-th district and in the *k*-th age group,respectively. *C* is a contact frequency matrix based on different districts (*R*) and different age groups (*A*), with the entries being *c*_*st*_ (where *s*, *t* ∈ {1, …, |*R*||*A*|}). We adopt the contact matrix in [50]. The contact patterns of *C* are divided into two parts: the frequency of social contacts between different age groups in the same district and the frequency of social contacts across districts. These levels of frequency are derived based on a study of human daily contact activities [51].

The entries *c*_*st*_ of the contact frequency matrix *C* are calculated as follows:

$$c_{st}=c_{\left((i-1)*|A|+j,(h-1)*|A|+k\right)}$$ (11)

where *c*_((*i*−1)*|*A*|+*j*,(*h*−1)*|*A*|+*k*)_ denotes the contact frequency between the individuals in the *j*-th age group of the *i*-th district and the individuals in the *k*-th age group of the *h*-th district.

The reproduction number *R*_0_ is one of the most important parameters in the HSEIR-V epidemic compartmental. This parameter denotes the number of newly infected individuals caused by an infectious individual in a susceptible population [52] [53]. *R*_0_ can be estimated from the existence of an epidemic-free equilibrium, which represents the host population as it appears (or does not appear) during the process of infection. Unfortunately, the traditional definition of *R*_0_ does not satisfy the requirement of the HSEIR-V model, as the population in the HSEIR-V model is divided into several subpopulations based on districts and age groups. To solve this problem, we adopt the next-generation matrix technique and the centre manifold theory proposed by Diekman and Driessche [52] [53]. In general, the reproduction number *R*_0_ is redefined as the number of newly infected individuals caused by an infectious individual in a susceptible population at the epidemic-free equilibrium.

To calculate *R*_0_, we need to consider the infected compartment *D*, in which the newly infected individuals will emerge. In the HSEIR-V model the exposed compartment, *E*, and the infectious compartment, *I*, are both viewed as parts of the infected compartment, *D*, which is calculated as follows:

D=[E,I] (12)

Hence,

$$F=\left(\begin{array}{c} \frac{1}{\left|R\right|\left|A\right|}\sum_{h=1}^{\left|R\right|}\sum_{k=1}^{\left|A\right|}\Delta_{hk}\cdot I_{hk}\\ \varphi_{ij}\cdot \left(1-\alpha_{ij}\right)\cdot E_{ij} \end{array}\right)$$ (13)

$$V=\left(\begin{array}{c} \varphi_{ij}\cdot E_{ij}\\ \psi_{ij}\cdot \left(1-\beta_{ij}\right)\cdot I_{ij} \end{array}\right)$$ (14)

where

$$\Delta_{hk}=c_{\left((i-1)*|A|+j,(h-1)*|A|+k\right)}\;\cdot \frac{S_{hk}}{P_{hk}}\cdot \theta_{hk}$$ (15)

where F and V denote the rate of population transitions for newly infected cases and the rate of population transitions by all other means.

The reproduction number *R*_0_ can be estimated at the beginning of the epidemic. *d*_0_ = (0, 0) take the form of an equilibrium solution with *E* = *I* = 0, which is calculated as follows:

$$F=\left[\frac{\partial F}{\partial D}\left(d_{0}\right)\right]=\left(\begin{array}{cc} 0 & \Delta_{hk}\\ \varphi_{ij}\cdot \left(1-\alpha_{ij}\right) & 0 \end{array}\right)$$ (16)

$$V=\left[\frac{\partial V}{\partial D}\left(d_{0}\right)\right]=\left(\begin{array}{cc} 0 & \varphi_{ij}\\ \psi_{ij}\cdot \left(1-\beta_{ij}\right) & 0 \end{array}\right)$$ (17)

*R*_0_ can be calculated as follows:

$$R_{0}=\rho \left(F\cdot V^{-1}\right)$$ (18)

where

$$F\cdot V^{-1}\left(\begin{array}{cc} 0 & \frac{1}{\varphi_{ij}}\cdot \Delta_{hk}\\ \frac{\varphi_{ij}\cdot \left(1-\alpha_{ij}\right)}{\psi_{ij}\cdot \left(1-\beta_{ij}\right)} & 0 \end{array}\right)$$ (19)

As *ρ* denotes the spectral radius of the matrix, which is the largest of the eigenvalues of the matrix, *R*_0_ is computed as follows:

$$R_{0}=\rho \left(\frac{1-\alpha_{ij}}{\psi_{ij}\cdot \left(1-\beta_{ij}\right)}\cdot \Delta_{hk}\right)$$ (20)

From the above formula, it is observed that *R*_0_ is related to the infection rate Δ_*hk*_ and the recovery rate *ψ*_*ij*_, which describe the pathological characteristics of an epidemic, and *c*_((*i*−1)*|*A*|+*j*,(*h*−1)*|*A*|+*k*)_ depicts the pattern by which the infection spreads among the individuals in different regions and different age groups. The basic reproduction number *R*_0_ was around 1.2–1.4 at the beginning of the epidemic in the 2009 Hong Kong H1N1 epidemic [45].

Vaccination is one of the most effective strategies, as this method protects vaccinated human beings and reduces infection transmissibility. Adopting the suitable vaccine distribution strategy at the suitable point in the epidemic can provide antiviral immunisation to the vaccinated population and reduce the dynamic of an epidemic. How to select a prioritisation strategy for distributing the vaccine is one of the most important topics for decision making in epidemic control.

The selection of a prioritisation strategy is affected by three important factors, namely vaccine coverage, vaccine releasing time and vaccine distribution method during the vaccination process [38]. Vaccine coverage represents the number of individuals who are vaccinated in the vaccination process, which changes the structure of the host population. The vaccinated population that has antiviral immunisation is determined by the vaccine coverage. The effects of different levels of vaccine coverage are explored by using the HSEIR-V model. We use this model for learning how to determine the most suitable vaccine coverage under conditions of limited vaccine resources.

Vaccine releasing time means the time when the vaccine doses are distributed. Releasing time affects the composite structure of the host population at different stages of the epidemic process. We investigate the effects of vaccination at different times by using the HSEIR-V model. This model allows us to study the efficiency of adjustment in the composite structure of the host population at different stages in the spread of a virus.

The vaccine distribution method involves the system for distributing vaccine doses to different host populations with different infection vulnerabilities and different contact patterns in various regions and age groups. Different vaccine distribution strategies affect the composite structure of the host population, which further influences the dynamics by which epidemics spread.

In summary, the effectiveness of vaccination for controlling the spread of an infectious disease is related to the adjustment of the composite structure of the host population, which includes adjustments for the size of the population, for the population in different stages during the spread of an epidemic, and the adjustment of the population in different districts and different age groups. The vaccine coverage, vaccine releasing time and vaccine distribution method each have an effect on the adjustment of the composite structure of the host population.

We evaluate several vaccine distribution prioritisation strategies, named S1, S2, S3 and S4, with respect to the HSEIR-V model as it corresponds to the populations of different regions and age groups.

(S1) The vaccine distribution strategy based on the population size [39]. The S1 strategy indicates that the number of vaccine doses is distributed to each population group in proportion to its population size. S1 is the most popular prioritisation strategy for deploying vaccine doses to multiple geographic regions, as this approach minimises the contradictions among local governments. Unfortunately, S1 fails to satisfy the requirement for focusing on the regions where vaccination is needed most urgently, and this approach tends to involve distribution of vaccine doses to regions where they are not necessary.

(S2) The vaccine distribution strategy based on the contact pattern matrix *C*. According to this strategy, those individuals in the subpopulation with a higher contact pattern frequency ($\sum_{h=1}^{\left|R\right|}\sum_{k=1}^{\left|A\right|}c_{\left((i-1)*|A|+j,(h-1)*|A|+k\right)}$), as calculated by the contact pattern matrix *C*, should have more vaccine doses. S2 is the most intuitive strategy, as it takes the contact frequencies of individuals in different geographic districts and different age groups into account.

(S3) The vaccine distribution strategy based on the infection rate *θ*. In this strategy, more vaccine doses are distributed to the population groups with high infection rates.

(S4) The hybrid vaccine distribution strategy based on the infectious risk. As the evaluation of infectious risk takes into account the effect of the population size, the contact pattern matrix and the infection rate, this strategy is a hybrid vaccine distribution strategy that combines the first three strategies. A number of vaccine doses is assigned to each population group according to its infectious risk *ϕ*_*ij*_. If the infectious risk *ϕ*_*ij*_ is large, the individuals in the corresponding subpopulation have a high probability of being infected. It is therefore reasonable to distribute more vaccine doses to that corresponding subpopulation. S4 considers the infection risk and it has a high probability of being an effective strategy, as the spread of infectious virus depends on the infection risk.

In summary, we explore the effects of vaccine coverage and vaccine releasing time in our simulation. We investigate how to select a prioritisation strategy from the above several vaccine distribution strategies under conditions where both the number of vaccine doses and the vaccine releasing time are fixed.

## Related work

Recently, researchers have paid increasing attention to the factor of vaccination. Compared with other kinds of intervention strategies, vaccine deployment is one of the most effective strategies, as this method protects vaccinated human beings and reduces infection transmissibility. One of the important issues for decision-making in epidemic control is how to select a prioritisation strategy for distributing the vaccine under conditions of limited vaccine resources.

Many studies have investigated how to effectively apply limited vaccine supplies for homogeneous or heterogeneous populations. These studies can be categorised into two subtypes. Studies of the first subtype have attempted to investigate the effects of different vaccine distribution strategies on disease control [54]–[58]. For example, Hsieh [54] explored vaccination strategies by taking age groups and intervention measures into account. Ghosh et al. [55] investigated the effects of intervention strategies that combined antivirus measures and vaccination campaigns to control the disease. Matrajt et al. [56] investigated which vaccination strategies were optimal when started at various points in time during the process of an epidemic. Milne et al. [57] explored the effects of several vaccine deployment strategies, such as the split vaccination strategy, the reactive strategy and the pre-emptive strategy. They tested these strategies by applying them to an actual community consisting of approximately 30,000 people in a developed country. Cruz-Aponte et al. [58] investigated the effects of different vaccination strategies to prevent influenza outbreaks under conditions of limited vaccine supplies.

Studies of the second subtype have aimed to identify the optimal vaccine deployment strategies for distributing vaccines [59]–[62]. For example, Medlock et al. [59] optimised influenza vaccine distribution by using survey-based contact data and mortality data as reported during influenza pandemics. These authors [60] also explored the optimal vaccine distribution strategy to reduce influenza-related deaths and minimise hospitalisations. Araz et al. [39] considered both age groups and locations in designing a geographic prioritisation strategy to distribute pandemic influenza vaccines. Tuite et al. [61] studied how to select an optimal vaccine allocation strategy for distributing vaccine among different age and risk groups within the Canadian population. Liu et al. [38] designed a modified compartmental infection model to select optimal vaccine deployment schedules. Lee et al. [37] identified the optimal age-specific vaccination strategies corresponding to the amounts of vaccines available and the timing of vaccinations to mitigate the Spring 2009 A (H1N1) pandemic in Mexico. Shim et al. [62] identified the optimal vaccination strategies for different age groups in terms of individual self-interest versus the interest of the whole population in reducing the effects of H1N1 infection-mediated morbidity and mortality. In addition, network analysis, especially gene-disease relationship prediction [63]–[66], is important on public health.

In summary, most of the previous work on vaccination has focused on age-specific vaccination strategies. The research works that have taken the combined effects of vaccination strategies and segregation strategies into account are less common. Our previous work in [37] developed a modified compartmental infection model by considering vaccination efforts for several age-specific host populations, and investigated ways of deploying vaccine more effectively. We also explored how to identify the relative priorities of subpopulations in terms of vaccination and contact reduction [67].

Compared with our previous work in [37] and [67], our work presented in this paper incorporates a combination of age-specific and geographic district-specific vaccination strategies along with segregation strategies in a proposed HSEIR-V compartmental model for preventing the spread of infectious diseases.

## Conclusion

In this paper, we perform a brief survey of ways to incorporate different intervention strategies such as vaccination, antiviral prophylaxis and treatment, or area and household quarantines into models for controlling the dynamics of an epidemic. Our major contribution is the designation of a hybrid SEIR model, which is named the HSEIR-V model. This new model takes into account the factors of both age and geographic district while designating several hybrid vaccine distribution strategies. We perform a thorough investigation of the HSEIR-V model while incorporating hybrid vaccine distribution strategies by simulation. As a result, we obtain several conclusions. (1) The HSEIR-V model is suitable for simulating the spread of infectious virus, in particular the 2009 Hong Kong H1N1 swine-flu epidemic. (2) A hybrid vaccine distribution strategy based on assessment of infectious risk is useful for holding a balance between avoidance of controversy among local authorities and meeting the actual requirements for controlling the dynamics of the epidemic. (3) Suitable parameters are important for improving the effectiveness and efficiency of vaccination efforts. (4) The combination of the vaccination and segregation strategies is more effective than a single-focus intervention strategy for controlling the spread of an infectious disease.

In the future, we will incorporate human behaviour into the HSEIR-V model, and compare with more age-based and geography-based approaches. The human beings involved will make their own decisions to accept or reject the vaccine injections. We shall use the HSEIR-V model to further explore adaptive vaccine distribution strategies and investigate how to find the optimal vaccine distribution strategy by assessing vaccine efficacy under conditions of limited vaccine resources. We will adopt some parallel operations when the scale of the network data is large.
